# A proof-of-concept study exploring the effects of impulsivity on a gamified version of the stop-signal task in children

**DOI:** 10.3389/fpsyg.2023.1068229

**Published:** 2023-02-09

**Authors:** Ruth Gallagher, Klaus Kessler, Jessica Bramham, Martin Dechant, Maximilian A. Friehs

**Affiliations:** ^1^School of Psychology, University College Dublin, Dublin, Ireland; ^2^ZEISS Vision Science Lab, Institute for Ophthalmic Research, Tübingen University, Tübingen, Germany; ^3^UCLIC, University College London, London, United Kingdom; ^4^Lise-Meitner Research Group Cognition and Plasticity, Max Planck Institute for Human Cognitive and Brain Sciences, Leipzig, Germany; ^5^Department of Psychology of Conflict, Risk and Safety, University of Twente, Enschede, Netherlands

**Keywords:** response inhibition, gamification, impulsivity, ADHD, stop-signal task

## Abstract

This proof-of-concept study provides an appraisal of a remotely administered gamified Stop-Signal Task (gSST) for future use in studies using child sample. Performance on the standard Stop-Signal (SST) task has been shown previously to differentiate attention-deficit-hyperactivity-disorder groups from controls. As is the case with the SST, it was envisaged that those with greater impulsivity would perform worse than those with lower levels of impulsivity in the gSST. The potential advantage of the gSST is that it could be perceived as less monotonous than the original SST and has the potential to provide higher data quality in child samples, however future research will need to be conducted to determine this. The gSST was administered remotely *via* video chat to 30 child participants within a community sample aged 8–12 to investigate the effect of ADHD symptoms and intrinsic motivation on gSST performance. Qualitative data was collected based on feedback from participants to gain insight into how the gSST was received by participants. A positive correlation was observed between impulsive/hyperactivity and gSST performance, however there was insufficient evidence to suggest that impulsivity predicted performance. With regards to accuracy, results suggested that impulsivity level significantly predicted the rate of go-omission errors. No relationships were observed between intrinsic motivation inventory (IMI) subscales and performance or IMI and impulsivity. Nevertheless, mean IMI scores were overarchingly high for each of the IMI subscales, suggesting that regardless of performance and/or level of impulsive behaviour, the child sample obtained in this study demonstrated high levels of intrinsic motivation, which was supported by the predominantly positive subjective feedback provided by the child participants. The present study provides some evidence based on quantitative and qualitative results for the efficacy of gSST for use with children. Future research with a larger sample of children is warranted to examine how performance on the SST and gSST compare/differ.

## 1. Introduction

Impulsivity is a multifaceted behaviour which encompasses, for example, poor response inhibition and reduced ability to delay reward (Winstanley et al., 2004; MacKillop et al., 2016; Jauregi et al., 2018). Higher levels of reward delay impulsivity and poor response inhibition are two prevalent symptoms of attention-deficit-hyperactivity-disorder (ADHD, Alderson et al., 2007). Response inhibition is critical for adapting to the dynamic environment of everyday life, for example, the ability to stop at a junction to avoid a pedestrian (Friehs et al., 2021). Poor response inhibition may sometimes lead to disadvantageous responses to stimuli.

ADHD is attributed to symptoms of inattentiveness, hyperactivity, and impulsivity (Barkley, 1997). Impulsivity is often measured using psychometric self/parent-report scales (Patton et al., 1995; Reynolds et al., 2006). Response inhibition is better measured *via* motor responses such as in the Stop-Signal Task (SST, Logan and Cowan, 1984). SST performance has been shown to differentiate ADHD versus non-ADHD controls (Oosterlaan et al., 1998; Castellanos et al., 2006; Tillman et al., 2007). While SST scores alone are not sufficient to provide an ADHD diagnosis due to high interindividual and intraindividual variability, the task has been used alongside self-report measures of impulsivity/ADHD to reduce the effect of social desirability (Aichert et al., 2012; Hedge et al., 2018; Jauregi et al., 2018).

Literature surrounding the SST in studies using non-clinical samples is less consistent. While Rubia et al. (1998) found reduced response inhibition ability *via* SST performance in samples of non-clinical impulsive children, Kuntsi et al. (2001) found no observable differences in SST performance with regards to impulsivity. This could suggest that reduced inhibitory capacity may only be consistently observed in samples where impulsive behaviours meet ADHD diagnosis criteria.

In the conventional SST, participants must respond to arrows presented on a screen with corresponding arrows on their keyboard and withhold their response if presented with a stop-signal (e.g., a sound). SST stimuli are typically presented in black and white on a screen without interfering distractions (Lappin and Eriksen, 1966). While this highly controlled setting gives rise to very precise measurements of response inhibition ability, the results may not translate to response inhibition in everyday life. Additionally, the task requires a high level of concentration over an extended period of time (Verbruggen et al., 2019). As the task is not particularly engaging and highly repetitive, there is risk that a lack of participant motivation may affect the quality of data collected (Kirkwood et al., 2010; DeRight and Jorgensen, 2014). This creates a dilemma when administering the task on child populations, especially those with pre-identified deficits in attention and concentration (Tillman et al., 2007). This can result in participant drop out, reducing the overall sample representation as well as rendering the data collected less representative of participants’ true abilities (Lipszyc and Schachar, 2010). This risks the generation of erroneous interpretations as poor task performance may not necessarily reflect poor response inhibition (Heilbronner et al., 2009).

Gamification is the application of game design elements to improve participant motivation (Lumsden et al., 2016a; Warsinsky et al., 2021). Gamification has previously been trialled in cognitive rehabilitation training and clinical contexts (Lim et al., 2012; van der Oord et al., 2012; Armstrong and Landers, 2017). Gamified cognitive tasks can appear more realistic and may elicit more natural responses without reducing accuracy of the measure (Friehs et al., 2020).

Gamification may help to improve engagement and reduce dropout rates, which is imperative when carrying out research on hard-to-reach samples (Zhou and Fishbach, 2016; de Paiva Azevedo et al., 2019). However, research on the efficacy of gamification thus far appears mixed. Some studies have reported positive effects of utilising game elements to improve working memory and concentration (Dovis et al., 2011; Ninaus et al., 2015). However, it must be noted that gamified adaptions to cognitive tasks may contribute to alteration in task performance, thus impeding the standardisation of the original measure (Hawkins et al., 2012). Adding elements of extrinsic motivation, for example through a points system, may increase the desire to succeed, resulting in improved task performance (Lumsden et al., 2016b). This motivational impact may risk overinflating actual response inhibition ability (Delisle and Braun, 2011; LePelley et al., 2015).

Lumsden et al. (2017) compared attrition and motivation levels when engaging in the standard SST to that of an intrinsically motivating and extrinsically motivating SST. The intrinsic condition replaced arrows with fruit and included a themed narrative, while the extrinsic condition used a points system. The intrinsically motivated themed game resulted in poorer performance and was rated as less enjoyable than the extrinsic game, suggesting that the intrinsic theme was more distracting and less motivating than the extrinsic condition. Further, there was no difference in level of attrition among the three conditions. Poorly implemented gamification techniques focusing more on the clinical effectiveness than motivation can impact game enjoyment (Khaleghi et al., 2020; Wiley et al., 2020).

More recently, Friehs et al. (2020) developed a gamified SST (gSST), including a themed premise, graphical elements of an avatar and fairy and an animated background (Nicholson, 2013). The gSST was purposefully developed to run closely to the standard SST. However, participants were instructed to follow the directions of the fairy to make their way out of the haunted forest into which they were lured by an evil witch. In contrast to Lumsden et al. (2017), the SST and gSST were administrated in a controlled lab environment which provided more control over task standardisation, the experimental set-up, and participant compliance. The haunted forest narrative may have generated an increased sense of urgency and determination to succeed than merely sorting fruit (Friehs et al., 2020).

This validation study has shown promising results, whereby the gSST was rated as more enjoyable than the SST without compromising on the task’s internal validity, which provides support for the gSST as an accurate measure of response inhibition. Results demonstrated that using a more visually complex and immersive task environment is useful in enhancing the ecological validity of the task, which is necessary to alter participants’ affective states, rather than merely changing the aesthetics of the stimuli (Wiley et al., 2020).

The aim of the present proof-of-concept study was to administer the gSST on a community sample of children aged 8–12 for the first time in order to ascertain whether future research with the gSST in child samples would be viable. As the gSST has not been previously administered with children before, level of burden to participants was kept to a minimum so as not cause unwanted fatigue. In doing so, only the gSST was administered, meaning there was no control measure (comparison results on gSST with SST). Nonetheless, the impact of ADHD behaviours and intrinsic motivation on gSST task performance were examined. As such, it was investigated whether impulsivity level was related to the degree of intrinsic motivation when engaged with the gSST. In addition, any feedback provided by participants throughout gSST administration was collected to gain insight into the experience of playing the gSST from the perspective of children.

## 2. Methodology

### 2.1. Overall research design

Parents along with their child were contacted *via* video chat using a laptop or computer. Firstly, the parent/guardian would provide brief demographic details followed by completion of the Barkley ADHD symptom scale on behalf of their child (Barkley, 2011). Participants were then provided with gSST instructions/narrative before taking part in 10 practice trials in order to gain familiarity with the go-stimuli and stop-signal stimuli. Child participants then completed 200 trials on the gSST, including 50 randomised stop-signal trials, taking approximately 12 min per child (Verbruggen et al., 2019). Approximate timing of the task and appropriate wording was determined *via* a pilot study with three participants. Upon completion of the gSST, child participants completed an adapted Intrinsic Motivation Inventory in order to gauge their motivation during the task (IMI; Ryan and Deci, 2000).

Participant feedback was recorded during the video call in order to facilitate improvement of the methodologies used in this proof-of-concept study for further research using the gSST with child cohorts.

### 2.2. Participants

Data were obtained from 30 children aged between 8 and 12 (*M_age_* = 10.033 years; *SD* = 1.47; 17 males and 13 females) and one of their parents. Four parents indicated that their child had a diagnosis of ADHD, the remaining had no ADHD diagnosis. Type of ADHD diagnosis, where applicable, was not recorded in the sample. Participants were recruited *via* posters and information sheets circulated by teachers/principals in five primary schools around Ireland to parents of children within this age range. Child participants did not require an ADHD diagnosis in order to take part. Children with ADHD were, however, eligible to take part so as to capture the variability of impulsivity within a community sample and four of the children (out of 30) had a diagnosis of ADHD.

### 2.3. Data exclusion

gSST data were excluded from participants who failed to meet criteria as per Verbruggen et al. (2019) and Verbruggen and Logan (2015). Two participants were excluded as they failed to inhibit any responses in the stop-signal trials, one participant displayed strategic behaviour during the gSST (e.g., waiting for the stop-signal to appear) and data obtained for one participant were removed as their overall go-trial accuracy rate was suboptimal. The remaining 26 participants were used in all reported analyses.

### 2.4. Gamified stop-signal task design

**Figure 1:**
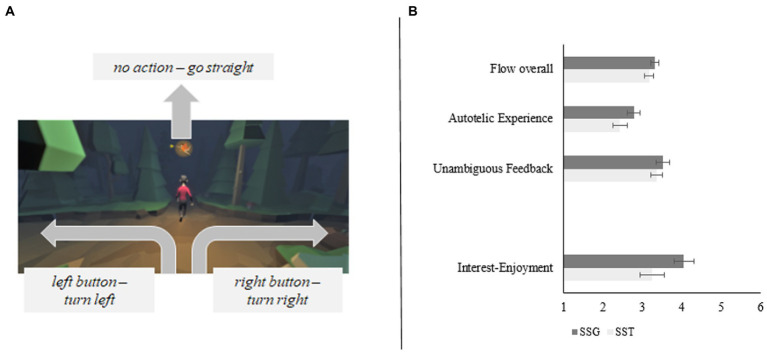
**(A)** Visual representation of the gSST. A fairy points the way to the left or right. The middle of the screen shows the running avatar. **(B)** Subjective differences in experiences for SSG and SST. The game-version led to a more enjoyable experience. All displayed differences were significant with *p* < .05. Figure adapted from Friehs et al. (2020), JMIR:Serious Games.

The gSST was built using the Game Engine by Unity3D (version 2019.01), in which participants were presented with an avatar running down a path towards a series of crossroads. Participants were instructed to use their keyboard arrow keys to follow the directions given by a magical fairy who would point her arrow in either the left or right direction in order to guide the avatar out of the haunted forest. The narrative describes an evil witch who masqueraded as the fairy, who can be identified *via* the auditory stop-signal. In cases where presentation of the fairy’s arrow was followed by the stop-signal, participants were told to withhold their keyboard response in order to avoid being lured deeper into the forest by the evil witch. See Figure 1 visual representation of the gSST as well as a comparison of subjective experiences between SST and gSST.

The gSST consisted of four blocks of trials, each containing 50 trials, 75% of which were go-trials and 25% were stop-trials. Between separate blocks, a pause of 15 s was given to allow the participant to get ready for the next block. The go-stimulus was presented for a maximum of 1,500 msec or until a response was produced. The stop-signal sounded over the laptop/computer speakers. Retaining the integrative method of the Horse Race Model (see Verbruggen et al., 2019), on successful stop-signal trials, the stop-signal delay (SSD) would be automatically increased by 50 ms in subsequent stop-signal trials and on unsuccessful stop-signal trials, the SSD would automatically decrease by 50 ms. The greater the SSD the more challenging effective response inhibition becomes. The intertrial interval was set to a random value between 500 msec and 1,500 msec.

### 2.5. Barkley ADHD symptom scale

The Barkley symptom scale (*a* = 0.961) is based on an 18-item DSM-5 ADHD symptoms checklist. Parents were asked to rate whether their child experienced each symptom on a scale of 0 (never) to 3 (always). Nine items related to inattention and nine items related to impulsive/hyperactivity. Of the impulsive/hyperactivity items, three related to impulsivity alone. This scale does not have a cut-off for ADHD diagnosis, so it was not possible to classify children as ADHD or non-ADHD (Barkley, 2011).

### 2.6. Intrinsic motivation inventory

The IMI is a 23-item scale (*a* = 0.688) consisting of 4 subscales: Enjoyment/interest (*a* = 0.864), perceived competence (*a* = 0.807), effort/importance (*a* = 0.588) and pressure/tension (*a* = 0.750; Ryan and Deci, 2000). Each item was rated through agreement with a statement on a 7-point scale. Higher scores indicated greater agreement with the statement. IMI subscales have previously been used in studies on children aged 12+ (Ostrow and Heffernan, 2018; Liu, 2021).

### 2.7. Data analysis

The SSRT was the dependent variable used as a determinant of response inhibition ability. Using the staircase integration method as proposed by Verbruggen et al. (2013), the SSD was continuously adjusted so as to obtain a probability of responding correctly [p(response|signal)] to approximately 50% of trials. Furthermore, two types of errors were recorded; go-omission errors, which indicate the rate of missed responses on go-trials and go-commission errors which demonstrate an incorrect direction of response on go-trials. Additionally, go-trial reaction times, indicating the speed of correct responses on go-trials, and stop-trial reaction times, denoting the time taken when incorrectly responding to stop-signals, were calculated.

A Python script (Version 3.10) was used to extract the gSST data and exported to IBM SPSS statistics (Version 27). The gSST data was matched with the survey data obtained *via* QualtricsXM Software for correlation and regression analyses.

## 3. Results

### 3.1. Demographics

There were no significant differences in performance with regards to gender [*t* (24) = −0.104, *p* = 0.918] or age [*F* (4, 21) =1.719, *p* = 0.183]. There were no observable difference in SSRT scores between those with and without an ADHD diagnosis [*t* (24) = 0.245, *p* = 0.809]. See Table 1 for an overview of the descriptive statistics for each of the three ADHD scale subscales.

**Table 1:** An overview of the mean scores obtained on the Barkley ADHD Symptom Scale and IMI subscales.

Subscale	*Min*	*Max*	*M*	*SD*
Barkley ADHD Symptom Scale				
Inattentiveness	0	21	7.3	7.036
Impulsive/hyperactivity	0	22	6.88	6.49
Impulsivity only	0	8	2.30	2.28
Total ADHD scale	0	41	14.19	12.64
Intrinsic motivation inventory				
Interest/enjoyment	2.57	7	5.15	1.06
Perceived/competence	2.67	7	4.83	1.14
Effort/importance	2.6	7	4.95	0.99
Pressure/tension	1	5.4	3.08	1.26

9 items are included in the inattentive subscale and 9 items are included in the impulsive/hyperactive subscale, in which 3 items correspond to impulsive only alone; mean IMI scores are within a range of between 1 (really disagree) and 7 (strongly agree).

### 3.2. Preliminary analysis

In order to validate the stop-signal data obtained, a paired samples t-test was used to confirm that there was a significant difference between the mean go-trial reaction time and the mean stop-trial reaction time (failed response inhibition) as recommended by Verbruggen et al. (2019). Higher response times were observed for go-trials [*t* (25) = 12.091; *p* = 0.005; *η*^2^ = 2.371] than stop-trials as expected for the gSST results to be validated on the present sample. See Table 2 below for details.

**Table 2:** Mean reaction times in seconds obtained for the performance measures used in gSST, overall accuracy score [p(response|signal)] and % rate of go-omission and go-commission errors in the sample.

Performance measure	*Min*	*Max*	*M*	*SD*
Go-trial reaction time (GRT)	0.88	1.31	1.13	0.12
Stop-trial reaction time (SRT)	0.77	1.25	1.04	0.131
Stop-signal delay (SSD)	0.12	0.82	0.596	0.223
Stop-signal reaction time (SSRT)	0.29	1	0.55	0.17
p(response|signal)	0.34	0.73	0.45	0.094
Go-omission error rate	0.01	0.326	0.13	0.082
Go-commission error rate	0	0.027	0.007	0.009

GRT is the average of the successful responses on go-trials; SRT includes the reaction time for erroneous responses on stop-signal trials; SSRT is the main performance measure; SSD is the average delay between the go and stop-signal; p(response|signal) indicates the overall mean accuracy rate which is optimal when the probability of responding on a stop trial is near 0.50. The % rate of go-omission and go-commission errors are relative to total trial count.

### 3.3. The relationship between impulsivity and gSST performance

**Figure 2:**
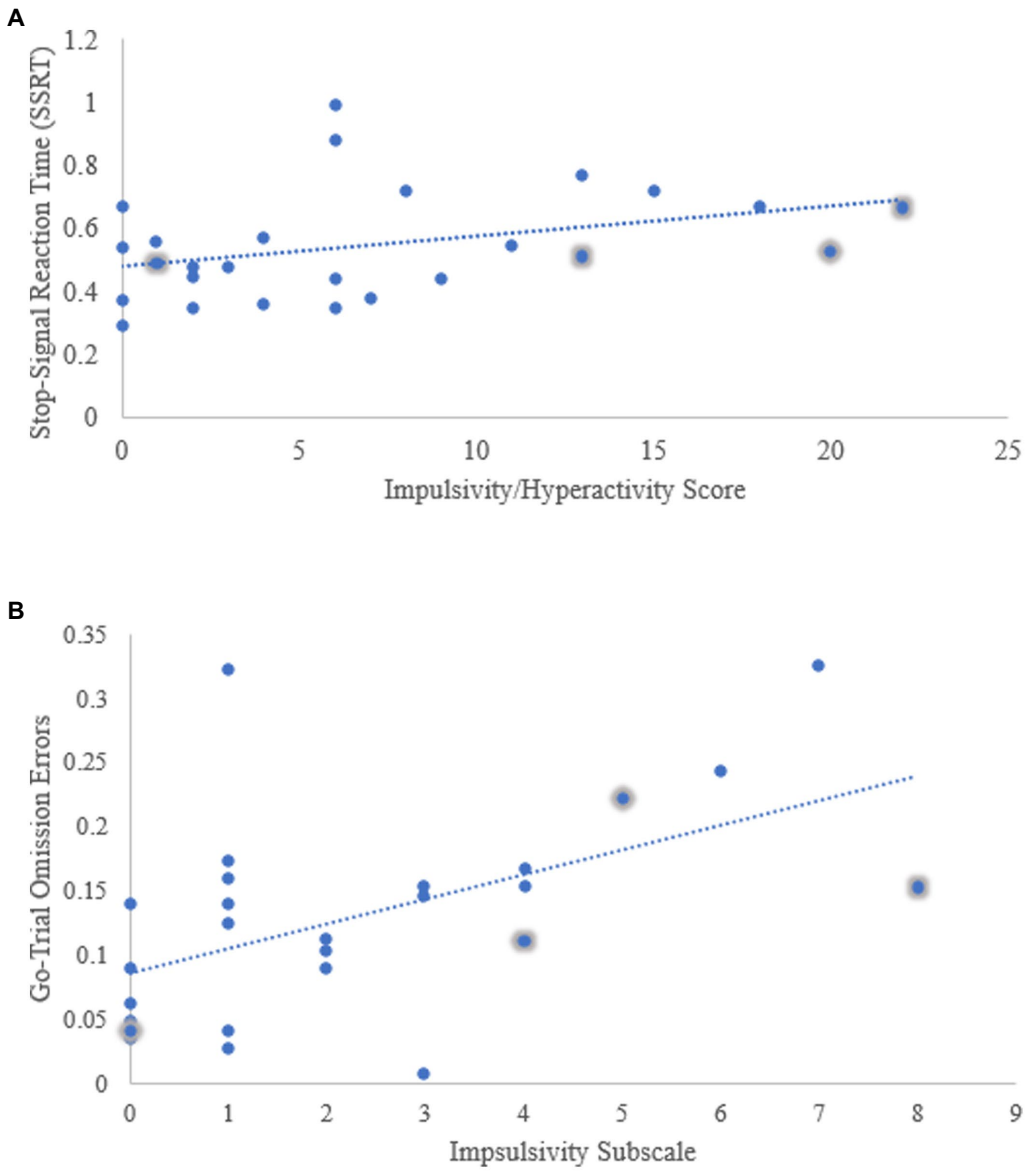
Scatter plots demonstrating **(A)**. The relationship between impulsivity/hyperactivity and gSST performance (SSRT) **(B)**. The relationship between impulsivity level and go-omission error rate.

The primary research question sought to explore the effect of impulsivity on gSST performance (SSRT). See Table 2 for an overview of the gSST performance data. A moderate strength relationship was observed between the impulsive/hyperactivity subscale and SSRT *r* (24) = 0.357, *p* = 0.037 as was expected based on previous findings, see Figure 2A. for a distribution of the scores. For the impulsive only subscale *r* (24) = 0.323, *p* = 0.054 and inattentive subscale *r* (24) = 0.179, *p* = 0.191, there were no significant correlations observed which does not support previous findings which suggest that impulsivity predicts performance on the gSST. As expected, there was no association between inattentiveness and gSST performance. Given that only one of the three subscales demonstrated a significant relationship with gSST performance, a simple linear regression model was produced to investigate the effect impulsive/hyperactivity behaviours had on SSRT scores. However, there was not enough evidence to suggest that level of impulsive/hyperactivity predicted gSST performance (*R*^2^ = 0.127; *F* (1,24) = 3.503, *p* = 0.073).

### 3.4. Error analysis

All three ADHD symptom subscales were analysed as predictors of go-omission error and go-commission error rate. For go-omission errors, the multiple linear regression model was statistically significant [*R*^2^ = 0.318; *F* (3,22) = 3.426; *p* = 0.035]. According to the model, it can be said that approximately 32% of the variance in go-omission error rate can be attributed to ADHD symptoms. Looking at the coefficients, it was found that the impulsivity subscale alone significantly predicted variation in go-omission error rate (*β* = 0.987, *p* = 0.045) while impulsive/hyperactivity (*β* = −0.544, *p* = 0.319) and inattentiveness (*β* = 0.075, *p* = 0.784) did not. Taking the adjusted *R^2^* into consideration, the model was strengthened by the removal of the inattentive subscale as a predictor from the model. (*R^2^*_inlcuding all 3 predictors_ *=* 0.226; *R^2^*_with 2 predictors_ *=* 0.257). See Figure 2B for a scatter plot demonstrating the distribution of go-omission error rate with regards to level of impulsivity.

The multiple linear regression model was repeated to examine whether ADHD symptoms predicted go-commission error rate. In this case, none of the three ADHD subscales of impulsivity, impulsive/hyperactivity or inattention [*R^2^* = 0.094; *F* (3,22) = 0.764; *p* = 0.526] were observed to significantly predict rate of go-commission errors made by respondents in this sample. It must be noted that there was a very low count overall of go-commission errors obtained within the present sample. See Table 2 for details.

### 3.5. The relationship between intrinsic motivation and gSST performance

There was no relationship observed between intrinsic motivation and gSST performance for any of the four subscales: Interest/enjoyment [*r* (24) = −0.082, *p* = 0.345], perceived competence [*r* (24) = 0.061, *p* = 0.383], effort/importance [*r* (24) = 0.036, *p* = 0.431] and pressure/tension [*r* (24) = 0.106, *p* = 0.304].

### 3.6. The relationship between impulsivity level and intrinsic motivation

There was no significant relationship observed between impulsivity with regards to intrinsic motivation: Interest/enjoyment [*r* (24) = −0.172, *p* = 0.200], perceived competence [*r* (24) = −0.234, *p* = 0.125], effort/importance [*r* (24) = −0.113, *p* = 0.292] and pressure/tension [*r* (24) = 0.208, *p* = 0.153]. See Table 1 for descriptive statistics.

### 3.7. Qualitative feedback

From a qualitative perspective, valuable feedback was provided about the gSST. Queries surrounding the appearance of the game became a standout theme. Some participants asked about introducing an element of urgency in the game, for example, being “*chased by the witch*,” if the *“forest was on fire”* or “*if there was a nuclear explosion*” to run away from. Others wanted to know if they could alter the appearance of the avatar “*Can I make the character* [Avatar] *look like me*?” and other elements of game appearance “*I do not like fairies, can I change the fairy to a monster*.” While there is risk that introducing an overarching sense of urgency to the game may drive feelings of extrinsic motivation rather than intrinsic motivation, there is some scope to introduce some of the suggested elements in order to retain motivation in the latter stages of the task to combat fatigue (Friehs et al., 2022).

At the end of the task, some participants were eager to know how well they had performed or if they had “*won*” the game. While some felt the task was challenging, they were reassured that the game was devised in such a way so to obtain individual success rates of approximately 50% and as a result it was normal to feel as though they had made errors. This theme was expressed frequently, as participants were prompted to reflect on their performance when completing the IMI. Such comments show the value of introducing performance feedback to participants either between blocks of trials or at the end of the task. Previous research shows that the feeling of successfully completing a task can fulfil certain needs which relate to intrinsic motivation as defined by the SDT, due to a feeling of competence (Ryan and Deci, 2000; Alexandrovsky et al., 2019).

Other participants mentioned that they “*did not think about wanting to do well* [during the game],” “*I just did my best*” while answering certain items on the IMI scale. This theme was interpreted as feelings of an inherent or intrinsic drive to do well. Furthermore, some participants struggled to reflect back on how they felt during the task, which may be an indication that they had reached some degree of flow, as described by Csikszentmihalyi (1990), while taking part.

## 4. Discussion

The present study sought to examine the gSST for use on a community sample of children aged 8–12. A moderate strength correlation was observed between the impulsivity/hyperactivity subscale and performance; however, impulsive/hyperactivity was not found to predict gSST performance. Furthermore, there was insufficient evidence to suggest that impulsivity alone related to gSST performance. While it is not known from the present study whether the strength of the relationship between ADHD symptoms and performance on the standardised SST would have been comparable (or even more pronounced given the monotony of the task), it is something worth assessing in future studies with larger samples. That being said, previous studies have struggled to find differences in SST performance in samples of children who demonstrate impulsive/hyperactive behaviours compared with controls (Daugherty et al., 1993; Kuntsi et al., 2001). Future research, including defined groups of children with and without a diagnosis of ADHD would be beneficial in order to investigate if the gSST has the same ability as the SST to differentiate between ADHD children and their neurotypical counterparts (Oosterlaan et al., 1998; Castellanos et al., 2006; Tillman et al., 2007).

With regards to errors on the gSST, impulsivity alone predicted almost 32% of the variance in go-omission errors which bolsters previous response inhibition research on ADHD vs. non-ADHD samples (Wodka et al., 2007; Shen et al., 2014). Therefore, there is evidence to suggest that the gSST is sensitive to typical performance patterns observed in samples of people with increased levels of impulsive traits.

Impulsivity did not predict level of go-commission errors. However, prevalence of this type of error remained low in the sample. Future gSST studies should investigate differences in error rates when child participants are subjected to both the original SST and the gSST. While Friehs et al. (2020) found no differences in error rate between the two tasks, error count was very low overall within this sample of healthy neurotypical adult participants. Taking ADHD traits into account, it would be worth investigating whether increased intrinsic motivation contributes to a reduction in go-commission and go-omission error rate, therefore, providing more reliable and better-quality data.

The relationship between intrinsic motivation and gSST performance as well as the effect of impulsivity on intrinsic motivation were analysed, with no relationship observed for either construct. Despite this, overall high mean IMI scores were observed. Therefore, regardless of performance and/or level of impulsive behaviour reported, the present sample demonstrated high levels of intrinsic motivation during the gSST. Making loose inferences between the present IMI scores and those obtained in the validation study, intrinsic motivation was higher within this child sample, indicating that the gSST may be more aptly suited to child cohorts than adult cohorts (Friehs et al., 2020). However, caution must be maintained when interpreting these claims as the sample size used in the study was small and lacking in variation.

Feedback and recommendations from participants were collected about the gSST. Many useful insights were expressed, providing valuable perspectives and suggestions for task improvement. For example, providing some level of performance feedback to participants either during or upon completion of the gSST could contribute to improving feelings of competency. Such simple adaptions could be beneficial over others which may create an extrinsically motivated pull that could distract from what is essentially being measured.

### 4.1. Strengths

Remote administration of the gSST proved possible. Children took part in the game remotely from the comfort of their own home. Level of task understanding was satisfactory, with data from only 2 of 30 participants excluded from final analyses due to lack of understanding of task requirements. However, it is unknown whether remote administration had any effect on gSST performance, and it could be argued that the casual setting may have contributed to the rate of go-omission errors due to participant distraction. It is possible that the benefits may outweigh disadvantages, especially for those who tend to perform less well in pressured situations, and allow researchers to collect data from a wider, more representative sampling pool (Assmann et al., 2016; Poulton et al., 2022). However, future research is required to confirm these observations.

### 4.2. Limitations

In this community sample, type of ADHD was not accounted for, whether that be impulsive/hyperactive type, inattentive type, or combined type (American Psychiatric Association, 2013). As a result, participants with inattentive type ADHD may have been categorised alongside those with impulsive–hyperactive type ADHD, despite a high level of variation in symptoms. This could provide some insight into the lack of observable differences in gSST performance between those with and without an ADHD diagnosis. However, only 4 out of the 26 child participants used in data analysis had an ADHD diagnosis, and as a result, the size of the group would have been too small to produce any significant differences between groups of participants whether there was a diagnosis of ADHD or not.

Furthermore, it should be noted that the overall internal reliability of the IMI scale was suboptimal within the present sample. IMI subscales have previously been adapted for administration in child/adolescent samples to suit the context and age of the cohorts (Ostrow and Heffernan, 2018; Liu, 2021). Some adaptations were made in order to ensure that children who took part were able to comprehend fully the meaning of each statement. Confirmatory actor analysis was not possible on such a small sample size. Therefore, it was unable to determine how well each subscale represented its desired construct. However, when the scale was broken down and reliability analysis conducted on each subscale, only one of the four subscales were found to have suboptimal internal reliability.

Furthermore, there was no data recorded as to whether the participants in the sample were interested in/engage in online games, which may have biased the enjoyment of and performance on the gSST if the games they play test response inhibition. Future research using the gSST should take this into consideration.

### 4.3. Avenues for task improvement/future research

Future research is needed to validate the gSST in a child sample. The gSST has the potential for further optimisation in order to render the task more applicable for everyday scenarios. The qualitative feedback obtained from participants in the present study has provided insightful suggestions for improvement of game characteristics. For example, a follow-up study by Friehs et al. (2022) has already demonstrated promising results when administering the gSST where participants were able to customise elements of the avatar’s appearance which led to increased interest and feelings of self-relevance, and therefore heightened engagement with the task. This characteristic of the gSST has the potential to be applied in settings where improvement of response inhibition ability is desirable, such as within cognitive rehabilitation settings or other therapeutic settings in order to improve response inhibition ability through training (Wang et al., 2013).

However, it must be noted that while positive feedback was provided, it is not possible to ascertain whether the gSST was better received by the children than the original SST as they were not subjected to both tasks.

Further, future studies may aim to validate the gSST in a larger child sample, as well as administer the basic task version for comparative purposes. However, future researchers need to consider that children, in particular, are prone to experimental fatigue and must plan the study accordingly (e.g., with larger breaks between tasks or administration over multiple days and with in-person rather than online testing).

## 5. Conclusion

This proof-of-concept study has provided some evidence for the efficacy of the gSST for use with children through feedback provided from participants as well as high levels of intrinsic motivation observed.

## Data availability statement

The original contributions presented in the study are publicly available. This data can be found here: osf.io/p4b5y [DOI 10.17605/OSF.IO/GF3VX].

## Ethics statement

The studies involving human participants were reviewed and approved by University College Dublin Ethics Committee. Written informed consent to participate in this study was provided by the participants' legal guardian/next of kin.

## Author contributions

All authors listed have made a substantial, direct, and intellectual contribution to the work, and approved it for publication.

## Funding

MF was supported by the German Research Foundation (DFG; FR 4485/1-1) and the Max-Planck Society.

## Conflict of interest

The authors declare that the research was conducted in the absence of any commercial or financial relationships that could be construed as a potential conflict of interest.

## Publisher’s note

All claims expressed in this article are solely those of the authors and do not necessarily represent those of their affiliated organizations, or those of the publisher, the editors and the reviewers. Any product that may be evaluated in this article, or claim that may be made by its manufacturer, is not guaranteed or endorsed by the publisher.
